# A Visit to Philadelphia and New-York

**Published:** 1859-12

**Authors:** 


### EDITORIAL AND MISCELLANEOUS.

### A Visit to Philadelphia and New York.

(Continued from the November Number.)

Having failed to complete in the last number of the Journal, the rapid review of Medical incidents and personages with which we met during our recent visit to New York and Philadelphia, we promised to resume the snbject in the present number.

A few nights after reaching New York, we had an opportunity of attending a meeting of the Academy of Medicine—the weather, however, was so inclement, that the attendance of members was not very large. Learning that the contagiousness of Yellow Fever was the subject for discussion, we had anticipated great pleasure in being present, but in consequence of the absence of several venerable gentlemen, who strenuously held to the contagious character of the disease, and whose peculiar views were to be the subject of discussion and criticism, the regular question was postponed until the next meeting. Other subjects of interest, however, came up during the evening, but that which most interested us, was a discussion which arose upon a report from the section upon Materia Medica, detailing by direction of the Academy, the manner of preparing Queru's Cod Liver Oil Jelly, samples of which had been presented to the Academy by the Manufacturer.

This report was made the occasion for an effort to exclude every article of this character from the Academy. That this is the true policy of the Academy, we think is beyond a question, for even if the parties presenting articles for which they claim *peculiar* merits, do not succeed in securing a *recommendation*, they yet get the advantage of *advertising* through the reports of the proceedings of the Academy, taken down during its sittings by reporters for the city papers, which by the way, is in our judgment, a most extraordinary and non-professional proceeding. With most serious disadvantages, this plan of reporting the proceedings of the Academy has no other advantage to the body, than as affording the more ambitious members an opportunity of displaying themselves through the newspapers. What material difference there is between this mode of advertising one's self, and the publication by Physicians and Surgeons themselves, of their cases and operations in the news-papers, we are at a loss to understand. We had supposed it a settled question of ethics, until this visit to the "Medical Metropolis," that the only legitimate channel for the publication of professional reports was through the Medical Journals of the country, enough of which there are in all conscience (not less than five being issued in New York city itself,) to meet fully the demand in this particular.

But to return to the subject of discussion, we would remark, that in our judgment, any Medical man, and to a much greater extent any Medical organization, and especially a body occupying the position, and exerting the influence, belonging to the New York Academy of Medicine, is reprehensible in the highest degree for any special recommendation extended to any article for which the Manufacturer claims peculiar merits on account of a *concealed* or *exclusive* method of preparation, or which carries along with it a recommendation for special diseases, with mode of application or administration, by which means the character of the disease, and the treatment adapted to its relief is determined, not by the Medical man—who can alone be competent for such a task—but by the manufacturer and the patient, thus most clearly supplanting the physician, and as far as it goes, rendering his office entirely useless, and indeed ignoring his very existence. Looking upon the whole thing as so absurd, and so entirely inconsistent with the honor, dignity, and rights of the Profession, as well as with the welfare of the diseased, we must confess to the greatest degree of astonishment, when a prominent Fellow of the Academy, arose during the discussion, and gravely read a certificate accompanying this same Queru's Cod Liver Oil Jelly, (dated some time before,) lauding in high terms its merits, to which was attached a number of the names of the most prominent members of the Academy.

From the tone of the discussion, however, we are satisfied that the members of that highly honorable and useful body, which ought to be the most prominent supporters of the honor and dignity of the Profession, and exponents of Medical ethics in the United States, will soon see the error of their way in this matter, and no longer allow themselves to be made the tools of those whose business it is to degrade the Profession, until it is frittered away into the hands of the Apothecary, and the manufacturer of patent instruments and apparatus. There was, we believe, no definite action upon the subject at this meeting, but we hope soon to chronicle an entire change in this whole matter, by the uniform refusal to endorse any *special* remedy or apparatus, and the complete exclusion of Reporters—except for Medical Journals—from their hall. Before concluding our notice of this meeting of the Academy, we would express our high appreciation of the courteous attentions received upon this occasion, from a number of the members of the Academy, and especially its accomplished President, Dr. John Watson.

Among a number of prominent Medical gentlemen with whom we met in New York, and to whom we were indebted for special attention, we would mention Dr. Horace Green, who gave us ample opportunities of witnessing his treatment of affections of the throat and air passages. Dr. Detmold, the distinguished Surgeon, through whose kindness, directly and indirectly, we were furnished with most valuable facilities. Dr. John C. Dalton, Jr., the eminent Professor of Physiology in the College of Physicians and Surgeons, and the *great Physiologist* of America, in whose lectures upon the circulation, which were being delivered at the time of our visit to New York, we were greatly interested, and in this connection we would remark, that these lectures were taken down at the time by a Reporter, for the New York Monthly Review of Medical and Surgical Science, and are now being published in that Journal, and are alone worth double the subscription price. We should be pleased to refer in detail to the many interesting and conclusive experiments upon animals, illustrating the views of Dr. Dalton, but this would be inconsistent with our plan, and we must refer the reader to the full reports contained in the Journal above referred to, which is edited by Dr. Austin Flint, Jr., one of the most promising young Medical men in New York, a former pupil of Dr. Dalton, and now Professor of Physiology and Microscopic Anatomy, in the New York Medical College, to whom, and to his eminent and noble father, Dr. Austin Flint, Sr., the popular Professor of Clinical Medicine in the New Orleans School of Medicine, and author of some of the most valuable of our American Medical works, we were placed under great obligations for the most marked attentions and most valuable aid in using our time to the best advantage. Through the kindness of Prof. Flint, Jr., we were placed in charge of the boldest, and perhaps the best, Surgeon in New York, the distinguished Chief of the Surgical Department of that immense establishment, the Emigrant's Hospital on Ward's Island, to visit which, he accompanied us, where we had an opportunity of witnessing some exhibitions of his extraordinary skill in Surgery. We had exceedingly interesting visits to the City Hospital on Broadway, to Bellevue on East River, and to that most admirably arranged of all the Hospitals we have ever visited, St. Luke's, which is located "Up Town," the founding and support of which, is in the highest degree creditable to the Episcopalians of New York, by whom it has been established.

We left New York with great reluctance, but soon found ourselves greatly interested in the more familiar walks of the City of our Alma Mater, Philadelphia, the seat of the *great Medical Schools* of the country, and while we think, and must say, that the Hospital facilities are more extensive, and greatly more accessible and valuable to a visitor in New York, the great centre of Medical training is still in Philadelphia, where the opportunities of Clinical teaching are all that can be used to advantage by the mass of Medical Students.

For a considerable portion of the pleasure derived from the visit to Philadelphia upon our return we were indebted to our honored and distinguished friend, Dr. Samuel D. Gross, the Professor of Surgery in the Jefferson Medical College, and we presume hardly inferior as a teacher of that department, to any living man. He, however, needs no eulogium from us, besides his exalted reputation as a practical Surgeon, and his previous works, which had given him the highest position as an author, his recent great work, "Gross's System of Surgery" in two volumes, illustrated by nine hundred and thirty-six engravings, and containing nearly twenty-five hundred pages, comprehending a Philosophical, Diagnostic, Therapeutic and Operative view of this great Science, will add to his reputation, and probably place him at the very front and head of American Surgery. We had an opportunity of attending his admirable Clinic at the Jefferson Medical College, which has had added to it, a tower of strength, in his accession to the Surgical Chair. It was at this renowned Clinic—which has for so many years been one of the great attractions of this leading School of the country—that we had the pleasure of hearing our own Carolina Dickson, who is spending the evening of a laborious and most honorable and eminent professional life, as the Professor of the Practice of Medicine in this Institution. If the same good judgment is continued in the future selections of Professors, as has characterized this School heretofore, it must long continue to occupy a most prominent position among the Medical Colleges of the country.

Without being able to go further into the detail of the particulars connected with our return visit to Philadelphia, (the advantages and prominent Medical men of which are so much more familiar to the Profession than those of New

York,) we must yet acknowledge our obligations to Dr. Pancoast, the distinguished Prof. of Anatomy in the Jefferson Medical College, and to Dr. Butler, the gentlemanly and accomplished Editor of the Medical and Surgical Reporter, for courteous attentions which contributed to the pleasure and profit of our stay in Philadelphia. Having already grown tedious in what was only intended as a rapid glance at the more prominent incidents of the trip, we will merely remark in conclusion, that we returned from our visit of Medical observation, with the conviction more fully impressed upon our mind, that American Medicine is rapidly taking a position in every particular fully equal to that of the Old World, and with a not less decided conviction that the advantages now offered to the Medical Student in the United States, do not suffer by comparison with those of Europe.

